# Supplementary material for: Neuropeptides specify and reprogram division of labor in the leafcutter ant Atta cephalotes
Source: bioRxiv. 2024 Nov 8:2024.11.07.622473. Preprint. [Version 1] doi: 10.1101/2024.11.07.622473 (PMC11581030; doi:10.1101/2024.11.07.622473)
Supplement: Supplement 1 [file NIHPP2024.11.07.622473v1-supplement-1.pdf]

bioRxiv preprint doi: https://doi.org/10.1101/2024.11.07.622473; this version posted November 8, 2024. The copyright holder has placed this preprint (which was not certified by peer review) in the Public Domain. It is no longer restricted by copyright. Anyone can legally share, reuse, remix, or adapt this material for any purpose without crediting the original authors.

Supplementary Table legends.

Table S1. Results from gene ontology analysis of subcaste differential expression categories noted here. The 7th column designates the specific subcaste or group tested and represents GO terms enriched among genes biased to that subcaste/group.

Table S2. Average transcripts per million, homology, and results of differential gene expression testing for *A. cephalotes* data here. For DEG calls empty cells indicate genes that lacked data (typically read counts) for testing, while “NS” indicates those with non-significant (padj $\geq$0.05) p-values.

Table S3. Proteomics results showing precursor charge, product charge, and fragments ions from identified proteins.

Table S4. Interacting protein with NPA following FLAG immunoprecipitation.

Table S5. Gene ontology results from experiments perturbing neuropeptides here. The 7th column designates the specific perturbation and direction of tested DEGs and represents GO terms enriched among genes up- or down-regulated in the designated perturbation.

bioRxiv preprint doi: https://doi.org/10.1101/2024.11.07.622473; this version posted November 8, 2024. The copyright holder has placed this preprint (which was not certified by peer review) in the Public Domain. It is no longer restricted by copyright. Anyone can legally share, reuse, remix, or adapt this material for any purpose without crediting the original authors.

Table S6. Average transcripts per million, homology, and results from comparing nurse and forager *from H. glaber* as well as associated NPA application to cultured *H. glaber* astrocytes. For DEG calls empty cells indicate genes that lacked data (typically read counts) for testing, while "NS" indicates those with non-significant (padj $\geq$0.05) p-values.

Table S7. Results of all behavioral assays presented here.

Video availability: Example videos for every experiment and condition have been provided. We will upload all videos upon publication, but are currently attempting to find a suitable hosting site for public sharing of >500Gb of videos.

bioRxiv preprint doi: https://doi.org/10.1101/2024.11.07.622473; this version posted November 8, 2024. The copyright holder has placed this preprint (which was not certified by peer review) in the Public Domain. It is no longer restricted by copyright. Anyone can legally share, reuse, remix, or adapt this material for any purpose without crediting the original authors.

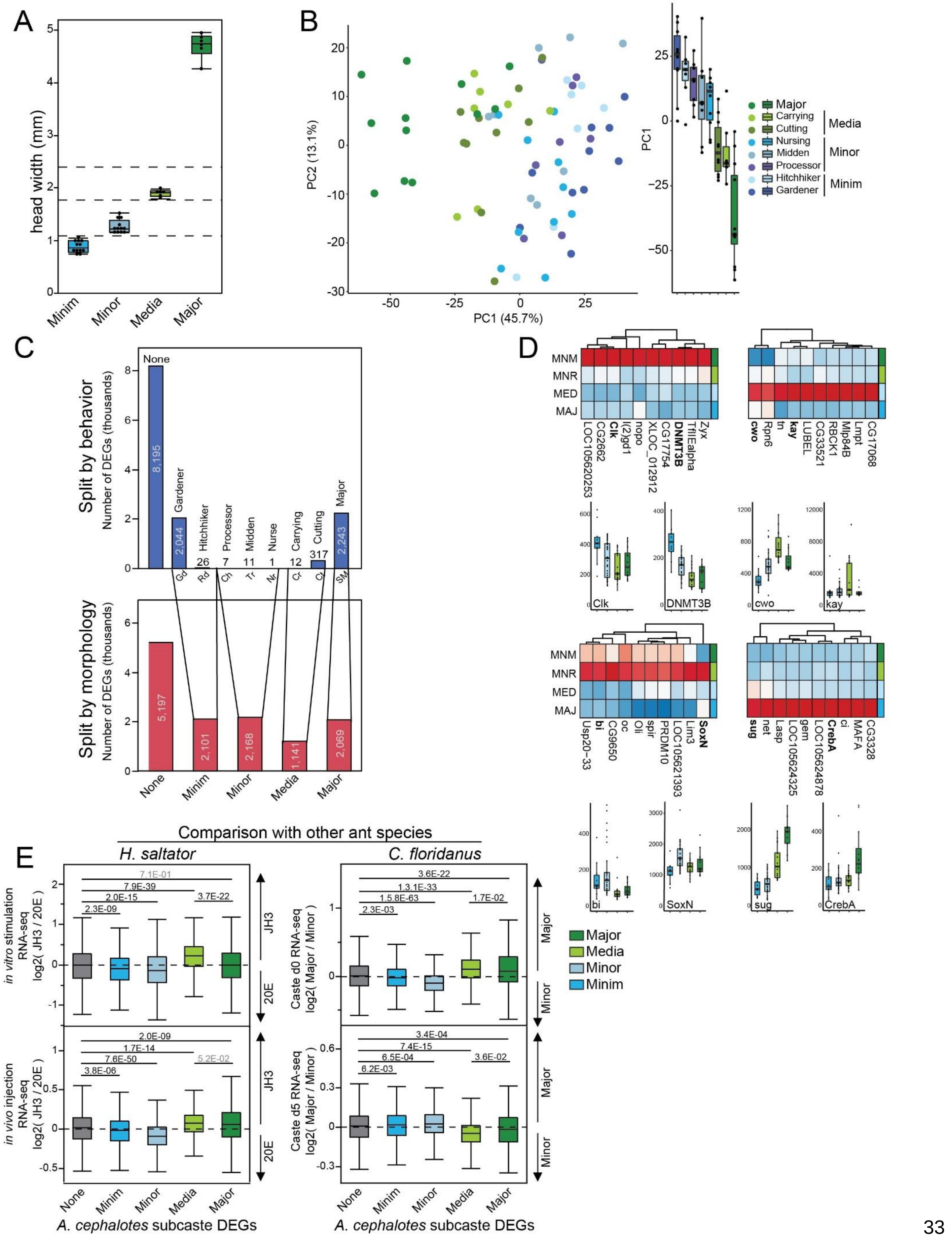

140

bioRxiv preprint doi: https://doi.org/10.1101/2024.11.07.622473; this version posted November 8, 2024. The copyright holder has placed this preprint (which was not certified by peer review) in the Public Domain. It is no longer restricted by copyright. Anyone can legally share, reuse, remix, or adapt this material for any purpose without crediting the original authors.

Figure S1.

A) Measurements of maximum head width for each of the primary worker subcastes as determined here.
B) PCA plot as in Fig 1B, but with all behavioral subcastes highlighted.
C) Comparison of genes significantly elevated in a given behavioral group (top) as compared to the respective worker morphometric subcaste, and associated numbers of genes significantly elevated in (top numbers) the behavioral groups and (bottom) the four morphometric subcaste groups. Overall comparing behavioral groups resulted in far fewer condition-biased DEGs, while comparing morphometric castes (lower) showed strong transcriptomic differences.
D) Top ten (by adjusted p-value from comparing each worker subcaste to all others) DNA binding domain containing genes significantly biased to each subcaste, with two representative genes' expression shown in boxplots below (normalized counts). Upper left: Top ten Minim-biased TFs, upper right: top ten Media-biased TFs, lower left: top ten Minor biased TFs, lower right: top ten Major-biased TFs.
E) Boxplots comparing genes biased to a given subcaste (relative to others) to log2-fold change of genes responding to JH3 or 20E in *H. saltator*[40] either (top left) *in vitro* or (bottom left) *in vivo*, as well as (right) log2-fold change from comparing *C. floridanus* Major and Minor worker brain transcriptomes. This illustrates comparisons with general signatures of JH3/20E (left panels), as well as differences between a binary worker subcaste system as seen in *C. floridanus* (right), which we have previously associated with JH3 signaling[7-9], overall suggesting persistent associations between worker caste and JH3/20E signaling in *A. cephalotes*. P-values generated by a Wilcoxon signed-rank test with Bonferroni correction following a significant Kruskal-Wallis test across all groups.

bioRxiv preprint doi: https://doi.org/10.1101/2024.11.07.622473; this version posted November 8, 2024. The copyright holder has placed this preprint (which was not certified by peer review) in the Public Domain. It is no longer restricted by copyright. Anyone can legally share, reuse, remix, or adapt this material for any purpose without crediting the original authors.

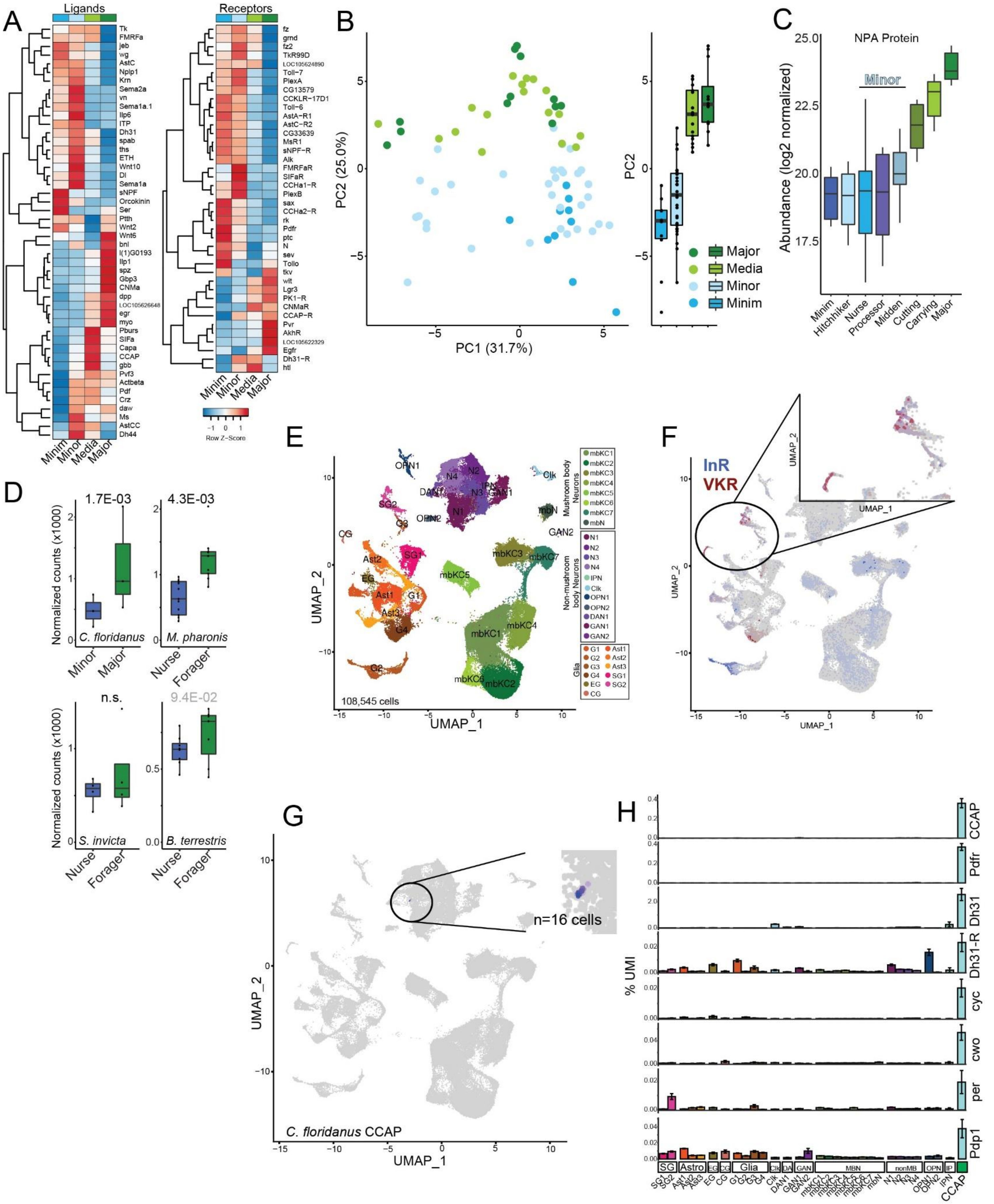

bioRxiv preprint doi: https://doi.org/10.1101/2024.11.07.622473; this version posted November 8, 2024. The copyright holder has placed this preprint (which was not certified by peer review) in the Public Domain. It is no longer restricted by copyright. Anyone can legally share, reuse, remix, or adapt this material for any purpose without crediting the original authors.

Figure S2.

A) More inclusive list of ligands and receptors (as compared to Fig 2B), not limited to neuropeptides or neuropeptide-like genes.
B) PCA analysis using only the genes presented in S2A (left) corresponding to peptide hormones, illustrating partial separation of subcaste based exclusively on this subset of genes.
C) NPA Mass Spectrometry protein abundance (n=3).
D) Normalized counts of NPA in four species showing significant or trending elevation in foraging subcastes in published data.
E) UMAP plot from *C. floridanus* scRNA-seq[9] illustrating cell types and cluster identities.
F) UMAP showing InR and VKR co-expression within *C. floridanus* brain scRNA-seq data, illustrating co-expression particularly among cortex glia and perineurial surface glia (inset).
G) UMAP plot showing cells expressing CCAP (expression > 4; 16 cells).
H) Genes showing highest expression within cells expressing CCAP relative to all other clusters in *C. floridanus* d0 scRNA-seq clusters, illustrating circadian and neuropeptide co-expression with CCAP

bioRxiv preprint doi: https://doi.org/10.1101/2024.11.07.622473; this version posted November 8, 2024. The copyright holder has placed this preprint (which was not certified by peer review) in the Public Domain. It is no longer restricted by copyright. Anyone can legally share, reuse, remix, or adapt this material for any purpose without crediting the original authors.

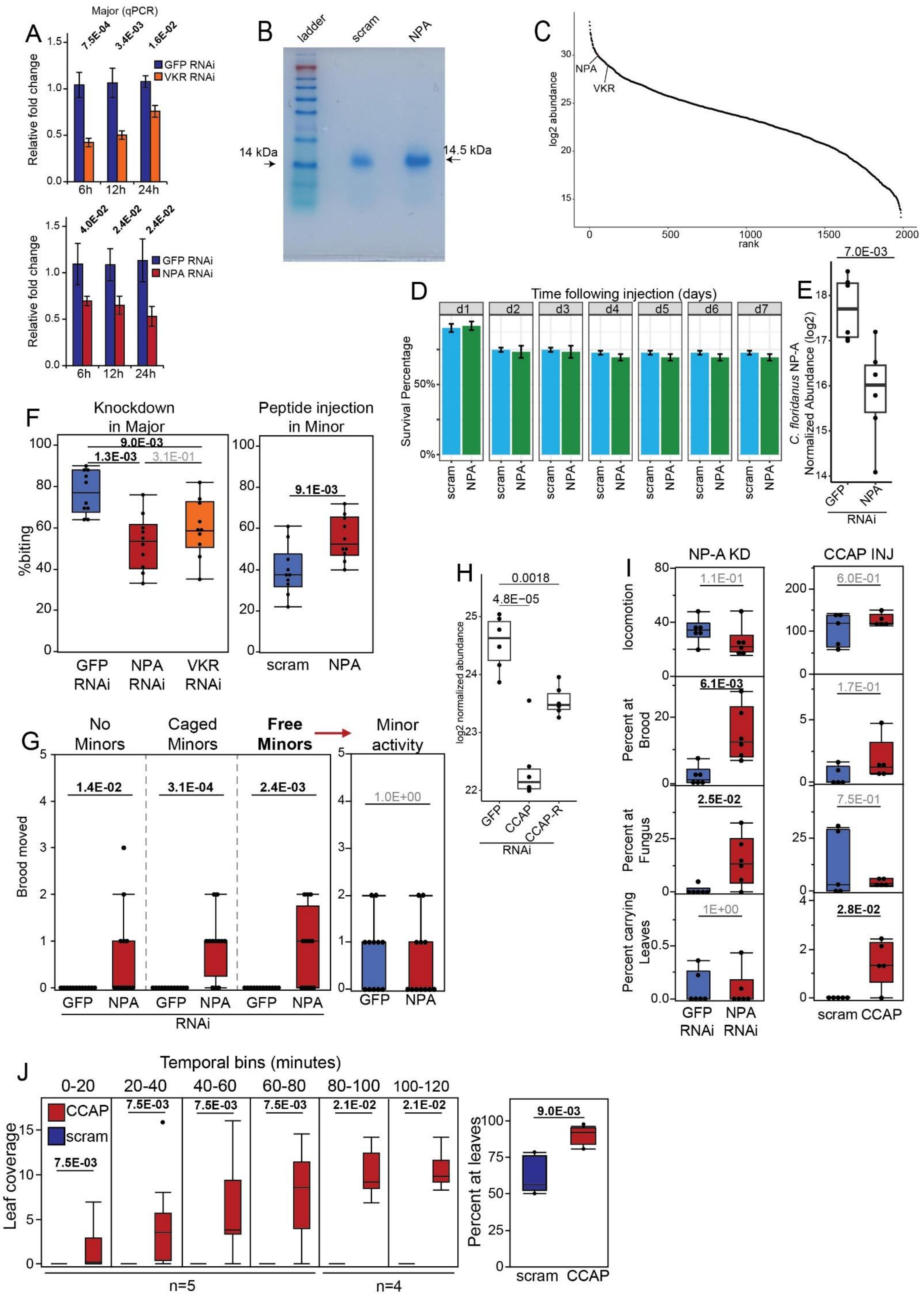

bioRxiv preprint doi: https://doi.org/10.1101/2024.11.07.622473; this version posted November 8, 2024. The copyright holder has placed this preprint (which was not certified by peer review) in the Public Domain. It is no longer restricted by copyright. Anyone can legally share, reuse, remix, or adapt this material for any purpose without crediting the original authors.

Figure S3.

A) KD validation for NPA and VKR via RT-qPCR (n=6, each sample type). P-values generated by a Mann-Whitney U test.
B) Coomassie gel to verify purification of scramble control peptide and recombinant NPA used here.
C) IP-MS of NPA from Major brain showing VKR as top interactor and only receptor Immunoprecipitated with NPA (n=3).
D) Mortality rates over 1-week for scramble control and NPA injection into brain (n=10).
E) KD at protein level of NPA in *C. floridanus* (n=3, each sample type). P-values generated by a Mann-Whitney U test.
F) Aggression assay (as in Fig 1B) performed for Majors following NPA or VKR KD as well as in Minor following NPA or scrambled control injection. NPA KD results in decreased aggression in Majors while NPA injection into Minors results in an increase in aggression. n=10 for all sample types.
G) Social context NPA experiment illustrating that the presence of air-exposed caged Minor workers (typical caretaking subcaste) or Minors free within the assay does not impact Major acquisition of caretaking following NPA KD. Right: Minor worker brood movement from the ‘free Minor’ assay show no difference in caretaking despite increased Major brood interaction. n=12, each sample type. P-values generated by a Mann-Whitney U test.
H) KD AT protein level of CCAP and CCAP-R (n=6, each sample type). P-values generated by a Mann-Whitney U test.
I) Results from automated video tracking of NPA KD Majors (left, n=6 for all sample types) and CCAP peptide injected Majors (right, n=5 for all sample types). P-values generated by a Mann-Whitney U test.
J) Binned automated assessment of Major leaf movement following CCAP peptide injection across 2 hours. Right: percent of time spent in leaf area overall for the same samples (n=5, each sample type). P-values generated by a Mann-Whitney U test of each pair.

bioRxiv preprint doi: https://doi.org/10.1101/2024.11.07.622473; this version posted November 8, 2024. The copyright holder has placed this preprint (which was not certified by peer review) in the Public Domain. It is no longer restricted by copyright. Anyone can legally share, reuse, remix, or adapt this material for any purpose without crediting the original authors.

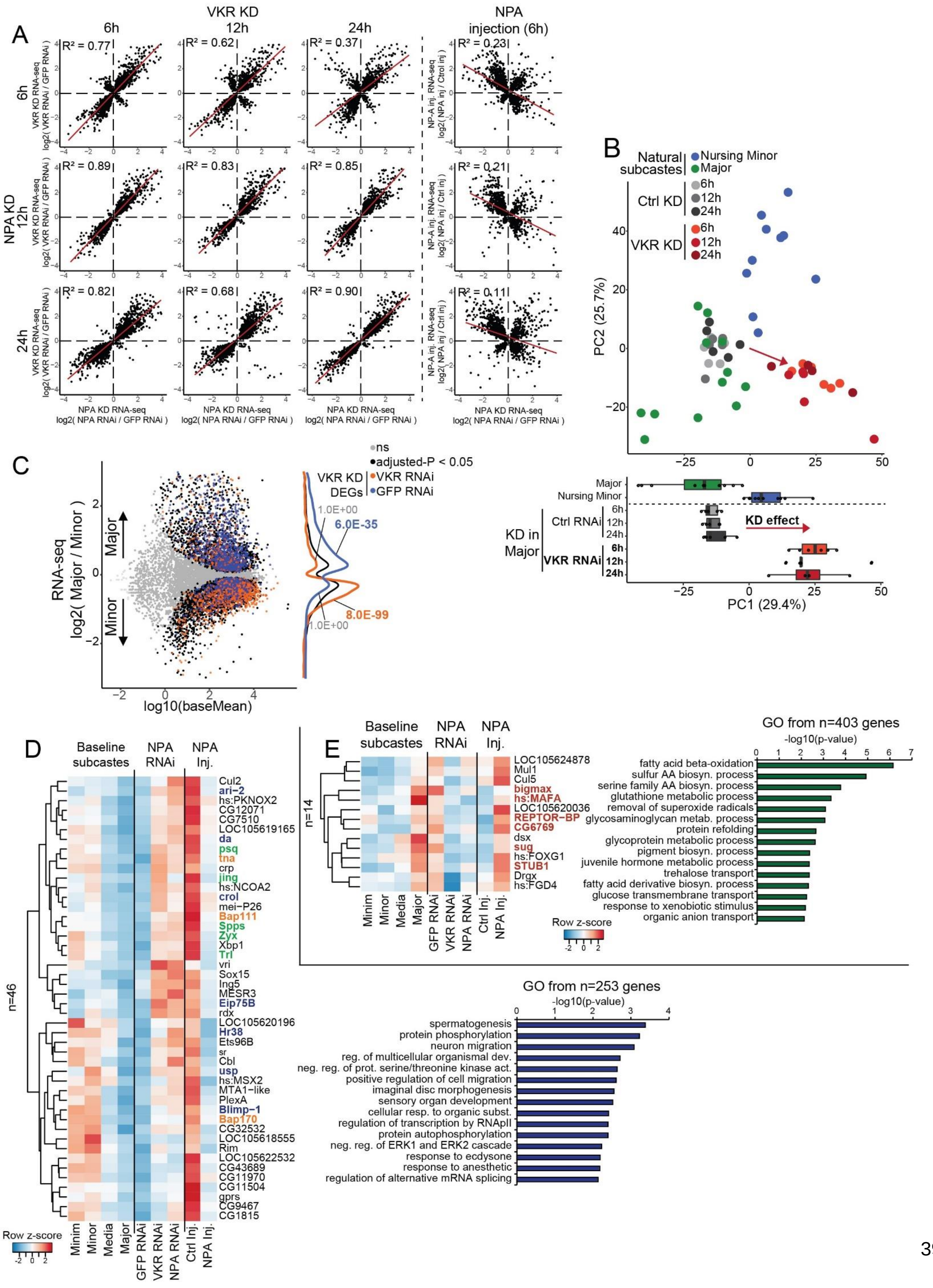

bioRxiv preprint doi: https://doi.org/10.1101/2024.11.07.622473; this version posted November 8, 2024. The copyright holder has placed this preprint (which was not certified by peer review) in the Public Domain. It is no longer restricted by copyright. Anyone can legally share, reuse, remix, or adapt this material for any purpose without crediting the original authors.

Figure S4.

A) Correlation scatterplots for NPA KD timepoints (rows) as compared to VKR KD timepoints (columns). R2 represent pearsons correlations between log2 fold changes for each comparison. Overall this illustrates strong correspondence between NPA KD and VKR KD across timepoints. Rightmost column: NPA KD timepoints compared to 6h NPA injection, illustrating negative association between perturbations as would be expected if lowering NPA and increasing NPA lead to similar transcriptomic outcomes.

B) PCA analysis of VKR (and GFP) KD samples as for Fig 4A.

C) VKR KD DEGs overlapped with *A. cephalotes* Major vs Minor DEGs, illustrating that VKR KD in Major results in up-regulation of genes biased to Minor and down-regulation of genes biased to Major, as for NPA KD (Fig 4A). P-values represent the results of a fisher's exact test comparing groups.

D) Heatmap of all genes with DNA binding domain-like annotation showing elevated expression in Minor, significant up-regulation upon NPA and VKR KD, and significant down-regulation upon NPA injection. right: GO terms associated with *all* genes showing this pattern (n=253). Genes with key functions in fly colored: orange: brahma complex; green: Polycomb related; blue: Ecdysone response genes.

E) Heatmap of all genes with DNA binding domain-like annotation showing elevated expression in Major workers, significant down-regulation upon NPA and VKR KD, and significant up-regulation upon NPA injection. right: GO terms associated with *all* genes showing this pattern (n=403). Red gene IDs represent genes related to regulation of energy homeostasis or metabolism.

bioRxiv preprint doi: https://doi.org/10.1101/2024.11.07.622473; this version posted November 8, 2024. The copyright holder has placed this preprint (which was not certified by peer review) in the Public Domain. It is no longer restricted by copyright. Anyone can legally share, reuse, remix, or adapt this material for any purpose without crediting the original authors.

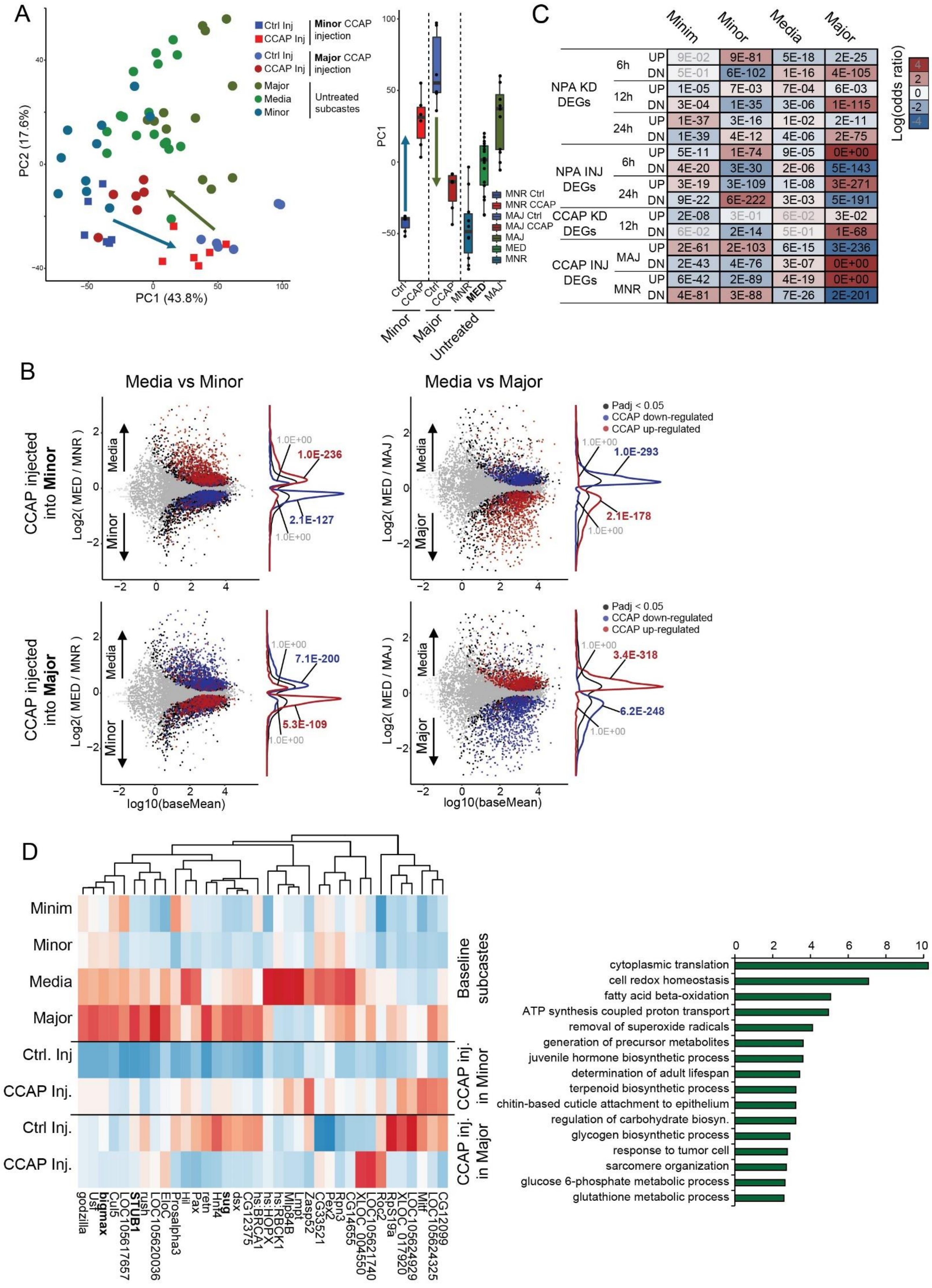

bioRxiv preprint doi: https://doi.org/10.1101/2024.11.07.622473; this version posted November 8, 2024. The copyright holder has placed this preprint (which was not certified by peer review) in the Public Domain. It is no longer restricted by copyright. Anyone can legally share, reuse, remix, or adapt this material for any purpose without crediting the original authors.

Figure S5.

A) PCA of result from CCAP injection in Major and Minor as compared to respective controls as well as representative baseline subcastes, illustrating that in Minor CCAP injection (squares) results in a transcriptomic shift “Media-ward” and in Major the same but from opposite ends of PCA space.

B) MA plots of baseline (left column) Media vs Minor and (right column) Media vs Major, overlain with (top row) Minor up- and down-regulated DEGs after CCAP injection, and (bottom roww) Major up- and down-regulated DEGs after CCAP injection into Major. Illustrating opposite effects that lead to up-regulation of genes typically elevated in Media, but only in the respective subcaste-of-injection. P-values represent the result of a fisher’s exact test comparing groups.

C) Odds ratio (colors) and p-values from overlapping of perturbation DEGs with subcaste-biased DEGs for all comparisons in *A. cephalotes* here. P-values present the results of a fisher’s exact test comparing groups.

D) The same as for S4D but using genes featuring a DNA binding domain-like annotation and showing higher expression in Media relative to Minor, and upregulation upon CCAP injection into Minor. Right: gene ontology enrichment for all such genes (n=1018), regardless of DBD domain. Bolded gene IDs are those mentioned in the text.

bioRxiv preprint doi: https://doi.org/10.1101/2024.11.07.622473; this version posted November 8, 2024. The copyright holder has placed this preprint (which was not certified by peer review) in the Public Domain. It is no longer restricted by copyright. Anyone can legally share, reuse, remix, or adapt this material for any purpose without crediting the original authors.

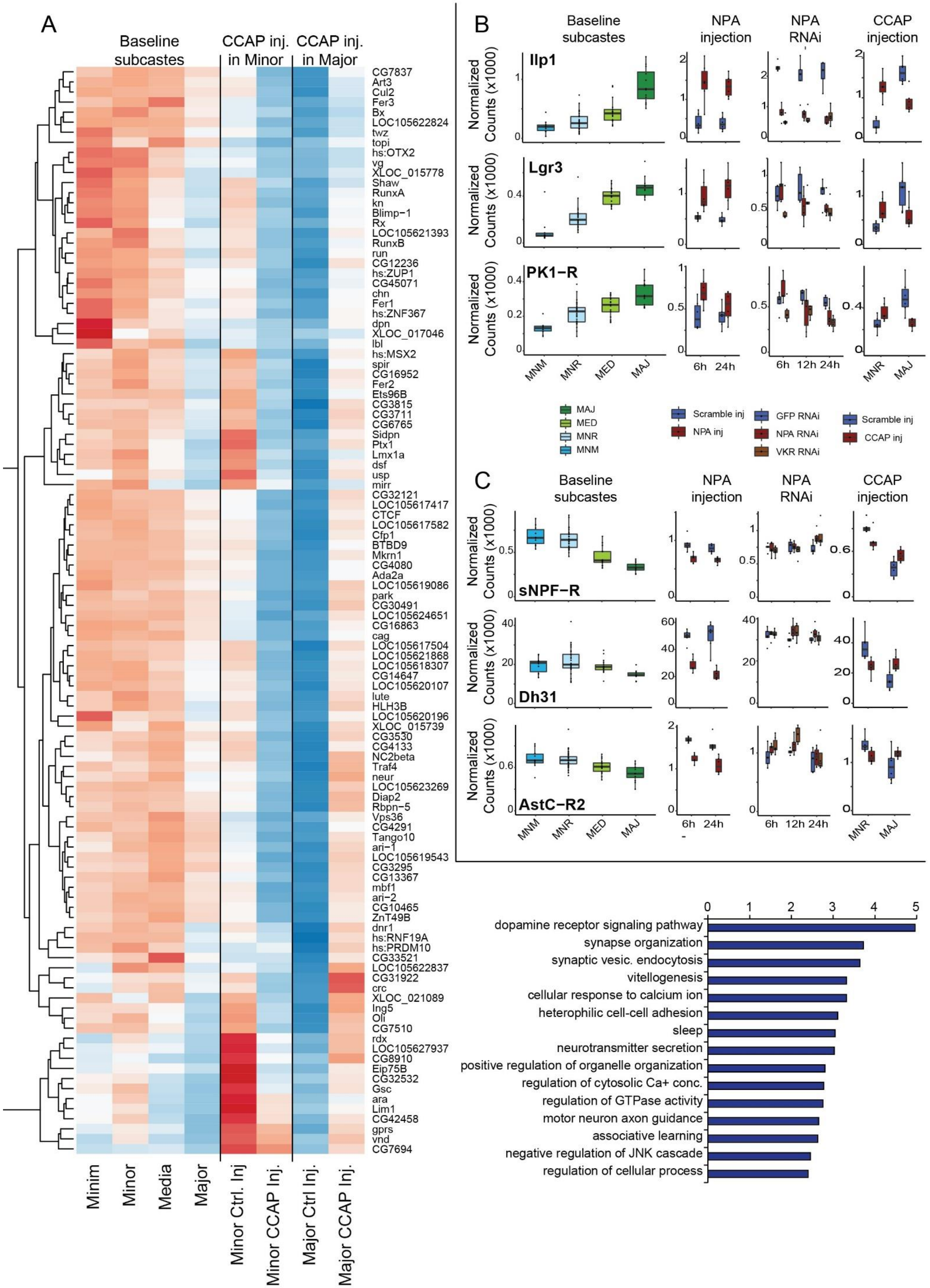

294

bioRxiv preprint doi: https://doi.org/10.1101/2024.11.07.622473; this version posted November 8, 2024. The copyright holder has placed this preprint (which was not certified by peer review) in the Public Domain. It is no longer restricted by copyright. Anyone can legally share, reuse, remix, or adapt this material for any purpose without crediting the original authors.

A) **Figure S6.** The same as for S4E but using genes featuring a DNA binding domain-like annotation and showing higher expression in Media relative to Major, and upregulation upon CCAP injection into Major. Right: gene ontology enrichment for all such genes (n=1,215), regardless of DNA binding domain-like annotation.

B) Normalized count plots (in thousands) of neuropeptides or receptors showing bias to baseline Major and consistent regulation by NPA injection (middle left), NPA KD (middle right) and CCAP injection in reciprocal subcastes (rightmost).

C) The same as S6B, but for those showing bias to baseline Minor/Minim.

bioRxiv preprint doi: https://doi.org/10.1101/2024.11.07.622473; this version posted November 8, 2024. The copyright holder has placed this preprint (which was not certified by peer review) in the Public Domain. It is no longer restricted by copyright. Anyone can legally share, reuse, remix, or adapt this material for any purpose without crediting the original authors.

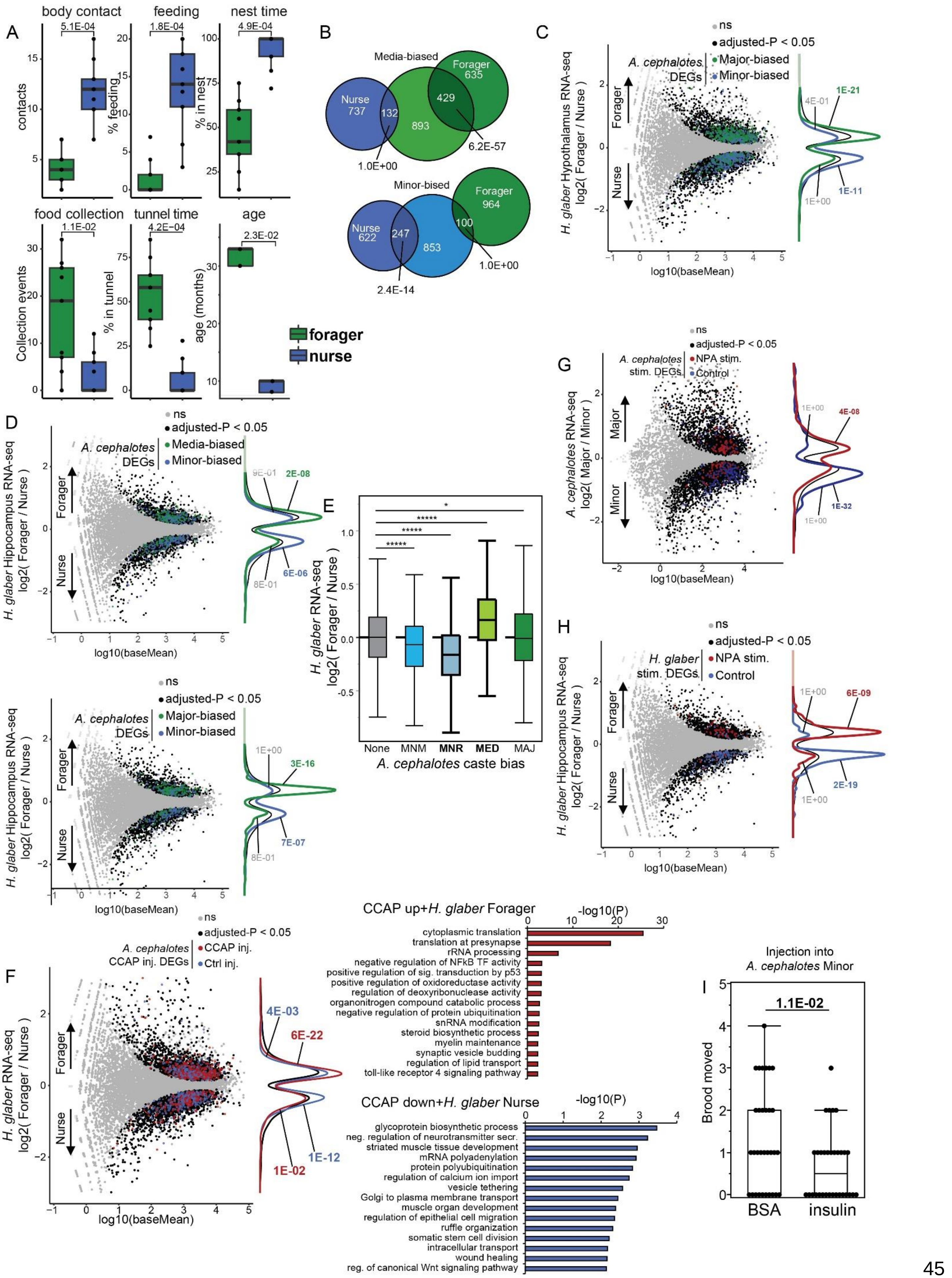

bioRxiv preprint doi: https://doi.org/10.1101/2024.11.07.622473; this version posted November 8, 2024. The copyright holder has placed this preprint (which was not certified by peer review) in the Public Domain. It is no longer restricted by copyright. Anyone can legally share, reuse, remix, or adapt this material for any purpose without crediting the original authors.

Figure S7.

A) Metrics distinguishing *H. glaber* Nurse and Forager. Nurses were determined based upon body contacts with other non-breeders, time spent feeding pups (higher in nurses) and time residing within the local nest. Foragers were determined based upon age, food collection activity and time in non-proximal nest tunnels. P-values generated by a Mann-Whitney U test, n=9. Each group but age for which n=3.

B) Venn diagram of the data shown in Fig 6B, showing numbers of genes. P-values represent the result of a fisher's exact test comparing groups.

C) Same comparison as for Fig 5B but overlain with **Major** vs Minor DEGs. P-values represent the result of a fisher's exact test comparing groups.

D) Same plots as figure 5B and S7B but for data from *H. glaber* Hippocampus, showing same trends. P-values represent the result of a fisher's exact test comparing groups.

E) Boxplots of *H. glaber* continuous subcaste bias (Forager / Nurse) as compared to major categories of subcaste genes in *A. cephalotes*, illustrating Minim and Minor genes show expression bias to *H. glaber* nurse, while Media show bias to *H. glaber* forager. P-values generated by a Mann-Whitney U test following a significant Kruskal Wallis test.

F) Overlap between CCAP injection DEGs upon CCAP injection into *A. cephalotes* Minor with *H. glaber* hypothalamus differential expression. Right gene ontology terms associated with each class of gene overlap. P-values represent the result of a fisher's exact test comparing groups.

G) Overlap between *H. glaber* Forager vs Nurse gene expression with DEGs from *H. glaber* astrocyte NPA stimulation. P-values represent the result of a fisher's exact test comparing groups.

H) Overlap between *A. cephalotes* Major vs Minor gene expression and up- and down-regualted genes following NPA stimulation of *A. cephalotes* neuronal cultures. P-values represent the result of a fisher's exact test comparing groups.

I) Insulin injection into *A. cephalotes* Minor brain shows reduction of brood movement behavior. Assay performed as for Fig 3B, but using insulin (or BSA as control) injection (n=32, each sample type). P-values generated by a Mann-Whitney U test.
